# An update on the secretory functions of brown, white, and beige adipose tissue: Towards therapeutic applications

**DOI:** 10.1007/s11154-023-09850-0

**Published:** 2023-12-05

**Authors:** Zeinab Ghesmati, Mohsen Rashid, Shabnam Fayezi, Frank Gieseler, Effat Alizadeh, Masoud Darabi

**Affiliations:** 1 Department of Medical Biotechnology, Faculty of Advanced Medical Sciences, Tabriz University of Medical Sciences, Tabriz, Iran; 2 Department of Molecular Medicine, Faculty of Advanced Medical Sciences, Tabriz University of Medical Sciences, Tabriz, Iran; 3 Department of Gynecologic Endocrinology and Fertility Disorders, Women’s Hospital, Ruprecht-Karls University of Heidelberg, Heidelberg, Germany; 4 Division of Experimental Oncology, Department of Hematology and Oncology, University Medical Center Schleswig-Holstein, Campus Lübeck, 23538 Lübeck, Germany

**Keywords:** Batokines, Brite fat, Extracellular vesicles, Lipocytes, Thermogenin, Transdifferentiation

## Abstract

Adipose tissue, including white adipose tissue (WAT), brown adipose tissue (BAT), and beige adipose tissue, is vital in modulating whole-body energy metabolism. While WAT primarily stores energy, BAT dissipates energy as heat for thermoregulation. Beige adipose tissue is a hybrid form of adipose tissue that shares characteristics with WAT and BAT. Dysregulation of adipose tissue metabolism is linked to various disorders, including obesity, type 2 diabetes, cardiovascular diseases, cancer, and infertility. Both brown and beige adipocytes secrete multiple molecules, such as batokines, packaged in extracellular vesicles or as soluble signaling molecules that play autocrine, paracrine, and endocrine roles. A greater understanding of the adipocyte secretome is essential for identifying novel molecular targets in treating metabolic disorders. Additionally, microRNAs show crucial roles in regulating adipose tissue differentiation and function, highlighting their potential as biomarkers for metabolic disorders. The browning of WAT has emerged as a promising therapeutic approach in treating obesity and associated metabolic disorders. Many browning agents have been identified, and nanotechnology-based drug delivery systems have been developed to enhance their efficacy. This review scrutinizes the characteristics of and differences between white, brown, and beige adipose tissues, the molecular mechanisms involved in the development of the adipocytes, the significant roles of batokines, and regulatory microRNAs active in different adipose tissues. Finally, the potential of WAT browning in treating obesity and atherosclerosis, the relationship of BAT with cancer and fertility disorders, and the crosstalk between adipose tissue with circadian system and circadian disorders are also investigated.

## Introduction

Recent extensive research has revealed that adipose tissue (AT) is not merely an inert fat storage tissue but also a complex organ that plays a fundamental role in controlling food intake, whole-body energy homeostasis, insulin sensitivity, blood pressure, angiogenesis, inflammation, and immunity by secreting various hormones and adipokines from different adipocytes [1–4]. AT is considered one of the largest complex endocrine organs in the body, with numerous potential therapeutic applications against obesity and related metabolic diseases [5]. It contains a complex mixture of cells, with one-third being mature adipocytes and two-thirds consisting of numerous cell types collectively known as the stromal vascular fraction. The latter fraction comprises a heterogeneous population of precursor cells and provides a rich source of mesenchymal stem cells (MSCs), which can be easily isolated from human AT. However, other cell types can be found in the fraction, such as pre-adipocytes, fibroblasts, endothelial progenitor cells (EPCs), pericytes, monocytes/macrophages, vascular smooth muscle cells, leukocytes, T-cells, and erythrocytes [6]. These cells form an effective communication network that regulates the activity and function of AT reservoirs.

Our previous studies have highlighted the importance of understanding the heterogeneity of AT and its impact on health [7–9]. Based on their color, ATs in mammals have been classified into three types: white AT (WAT), brown AT (BAT), and beige (brite, brown-in-white, brown-like) AT (beige AT). WAT is primarily involved in energy storage; whereas BAT is predominantly responsible for non-shivering thermogenesis, beige AT expends energy to generate heat during cold exposure (cold-induced thermogenesis). This review focuses on the differential properties of white, brown, and beige ATs, the molecular mechanisms underlying the development of adipocytes, and the significant roles of batokines and regulatory miRNAs in different ATs. In addition, it scrutinizes WAT browning by various agents as a therapeutic target against obesity and related metabolic disorders such as atherosclerosis, cancer, and fertility. Finally, the relationship of BAT with cancer and infertility and the crosstalk between AT, circadian system, and circadian disorders will be studied.

## White, brown, and beige adipose tissue

The expansion of WAT begins shortly after birth [10]. WAT is found throughout the body and is classified into two groups: visceral WAT (vWAT) depot and subcutaneous WAT (sWAT) depot [11]. Lineage tracing experiments have shown that white adipocytes arise from embryonic mesothelial cells, and the segregation of visceral and subcutaneous adipocytes occurs during late embryogenesis [12]. Morphologically, WAT is typically characterized by large unilocular/large lipid droplets, limited mitochondria, and ivory or yellowish color. WAT is mainly composed of unilocular adipocytes named white adipocytes [13]. Following overeating or low energy intake, WAT can capture free fatty acids (FFAs) and glucose from blood plasma and convert them into triglycerides (TGs). Adipose TG lipase (ATGL) catalyzes the first stage of lipolysis of cytoplasmic triacylglycerols in WAT [14, 15]. Therefore, WAT supplies energy during fasting and regulates energy homeostasis. WAT is highly innervated with afferent and efferent sympathetic nerves; but has no parasympathetic innervation [16]. In the case of obesity, hyperplasia, hypertrophy, secretion of vasoconstrictors, and immune cell infiltration are observed in WAT [17]. BAT is formed and differentiated before birth to protect newborns from cold [18, 19]. BAT is present and active in human adults, and its activity is decreased in various pathological states such as obesity, diabetes, and aging [20]. Human BAT is in the supraclavicular, axillary, neck, periaortic, paravertebral, perirenal, and mediastinal regions [5, 21]. In mice, BAT is mostly in the interscapular region [22]. In humans, BAT comprises a small portion of AT and vanishes from most parts of the body with age, remaining only around deeper organs [23]. BAT is considered to have a high degree of vascularization and sympathetic innervation [24]. Compared to WAT, BAT has significantly high mitochondrial content and macroscopically appears brown due to heme cofactors in the mitochondrial enzyme cytochrome oxidase [25]. BAT is mainly composed of brown adipocytes that contain large amounts of multilocular/small lipid droplets of various sizes. Brown adipocytes are small and polygonal, unlike white adipocytes [26].

**Fig. 1:**
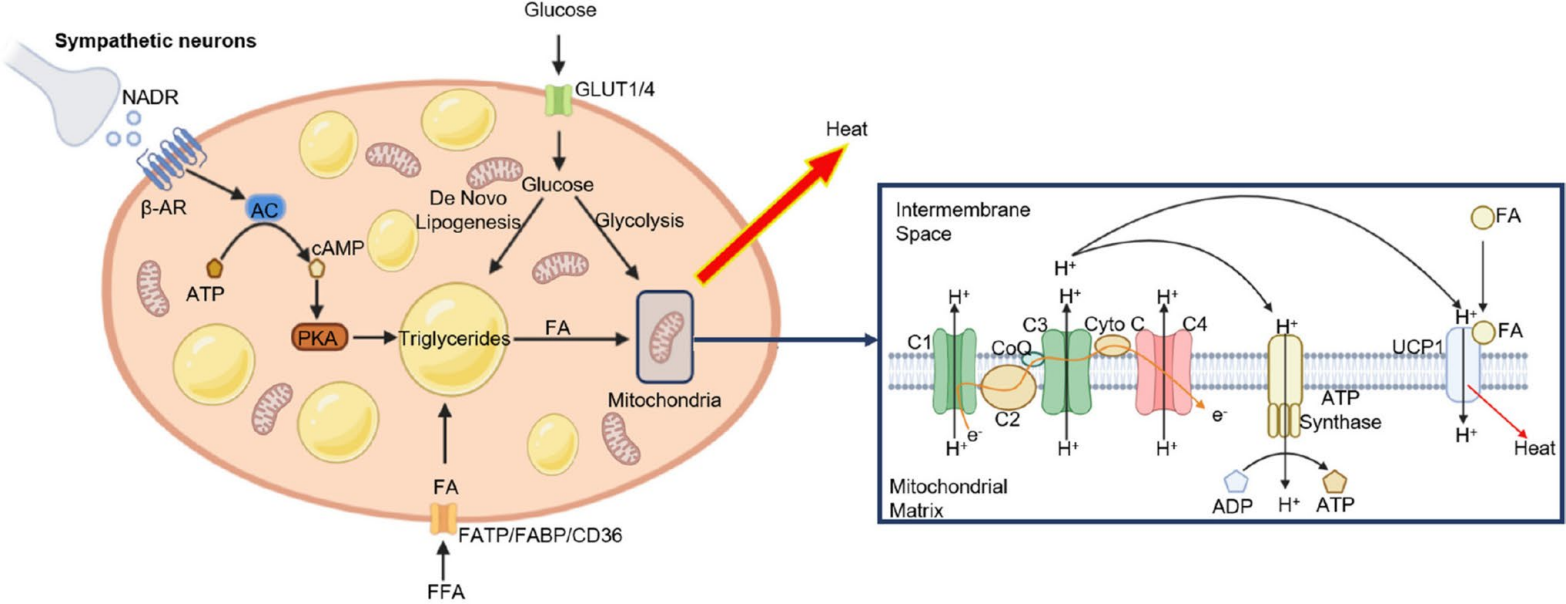
BAT activation and molecular mechanism of UCP1 function studied by McNeill et al. [27] (with permission). Upon exposure to a cold stimulus, sympathetic neurons release NADR from the synapse region. NADR attaches to several β-ARs on the brown adipocyte, activating adenylyl cyclase (AC) and converting ATP to cyclic adenosine 3′,5′-monophosphate (cAMP) which, in turn, causes protein kinase A (PKA) activation. PKA induces the lipolysis of TG stores and the release of fatty acids (FAs), which are the primary substrate for the thermogenesis process; these attach to and activate the UCP1 protein in the IMM. Utilizing the electrochemical proton gradient of the electron transport train, the UCP1 protein transports H^+^ ions across the IMM. This leads to uncoupling cellular respiration from ATP synthesis, releasing heat instead of ATP production. FFAs are transported into the brown adipocyte via fatty acid transport protein (FATP), fatty acid binding protein (FABP), and a cluster of differentiation 36 (CD36). Glucose is also transported into the brown adipocyte via the glucose transporters (GLUT1/4). Uptake of circulating FFA and glucose helps the regeneration of intracellular TG stores. Moreover, glucose substrate enters the tricarboxylic acid cycle (TCA, also known as the Krebs or citric acid cycle) via the glycolysis process. C1–4, complex 1–4; CoQ, co-enzyme Q; Cyto C, cytochrome C; e^−^, electron; H^+^, hydrogen ion

BAT preserves body temperature by a process known as non-shivering thermogenesis, dissipating energy in the form of heat. Thermogenesis is mediated through brown adipocyte-specific uncoupling protein 1 (UCP1), also called thermogenin, in response to adrenergic signaling via the sympathetic nervous system [27]. Upon exposure to a cold stimulus, sympathetic neurons release noradrenaline (NADR) from the synapse region. NADR attaches to several β-adrenergic receptors (ARs) (classically β3-AR but also β1- and β2-AR [28, 29]) on the brown adipocyte. Activation of β-ARs triggers a signaling cascade that leads to lipolysis of TG stores and release of fatty acids (FAs) and, finally, activation of UCP1 [27] (Fig. 1). UCP1 protein is located in the inner mitochondrial membrane (IMM) and transports H^+^ ions across the IMM in the presence of FAs and glucose, leading to the uncoupling of cellular respiration and ATP synthesis, thereby releasing heat instead of ATP production [27, 30] (Fig. 1). Thus, BAT plays a natural anti-obesity role. However, the precise mechanism of function of UCP1 is not well understood as direct methods are unavailable [31].

White adipocytes express several enriched genetic markers, including *LPL*, *G3PHD*, *ASC1*, *Tcf21*, *Hoxc8*, *Hoxc9*, and *TLE3* [32–34]. As a major endocrine organ, WAT also secretes hormones such as adiponectin, leptin, resistin, and cytokines such as tumor necrosis factor-alpha (TNF-α), IL-6, and monocyte chemotactic protein-1 (MCP-1), which play a role in regulating whole-body metabolism, insulin resistance, and low-grade systemic inflammation connected with obesity [35, 36]. In addition to *UCP1*, *Cidea*, *PPARA*, *Pgc1a*, *Prdm16* [36], *LHX8*, *Zic1* [33], *Eva1*, *Pdk4*, *Ebf3*, and *Hspb7* [37, 38] have also been described as BAT-specific genetic markers [20, 32]. UCP1 is nearly undetectable in WAT, but the UCP2 isoform is expressed in parts of WAT [39]. The main genetic markers for BAT and WAT are listed in Table 1.

**Table 1:** Principal differences among the three types of adipose tissue: white, brown, and beige

**Characteristic property**	**White**	**Brown**	**Beige**	**Reference (s)**
Location in humans	Subcutaneous, visceral	Supraclavicular, axillary neck, periaortic paravertebral interscapular, perirenal	Supraclavicular, cervical	[33, 48, 51, 52]
Location in mice	Gonadal, mesenteric, inguinal, retroperitoneal	Interscapular supraclavicular, cervical axillary, periaortic paravertebral, perirenal	Interscapular, anterior subcutaneous, inguinal WAT	[33, 52]
Morphology				[26, 53]
Shape	Spherical	Ellipsoid/polygonal	Spherical	
Cell size	Variable, large (25–200 μm)	Small (15–60 μm)	Variable, smaller than white	
Lipid droplets	Unilocular/Single large lipid droplets	Multilocular/ multiple small lipid droplets	Multilocular /multiple small LD with variable size	
Mitochondria	+	+++	++ (upon stimulation)	
Functions	Energy storage, endocrine (adipokines)	Energy expenditure (thermogenesis), anti-inflammatory, cardioprotective, endocrine (batokines)	Energy expenditure (adaptive thermogenesis), anti-inflammatory, cardioprotective, endocrine (batokines)	[53, 54]
Adipocyte progenitor cell markers	Myf5^+^, PDGFRα/β^+^, CD24^+^, CD34^+^, Sca1^+^, CD44^+^, VCAM1^+^, CD29^+^, Lin^−^, CD45^−^, CD31^−^, CD9^+^	Myf5^+^, Pax7^+^, En1^+^, Pax3^+^, Trpv1^+^, CD31^−^, CD45^−^, Prx1^−^	Myf5^−^, PDGFRα^+^, Sca1^+^, SMA^+^, CD81^+^, CD29^+^, Lin^−^, Pax3^−^, CD34^−^, MyoD^+^	[51, 55]
Iron content	+	+++	+++	[56]
UCP1 expression	Nearly undetectable	+++	++ (upon stimulation)	[48, 56]
Main thermogenic mechanism	-	UCP1 dependent	UCP1 dependent, creatine cycling, Ca^++^ cycling	[54, 55]
Vascularization and innervation	Low	High	High (upon stimulation)	[56–58]
α-, β-adrenergic receptors	β3 (++), α2 (+)	β3 (+++)	β3/α2?	[56]
Changes during obesity	Hyperplasia, hypertrophy, secretion of vasoconstrictors, high infiltration of proinflammatory immune cells	Potentially resistant to obesity-induced inflammation,	Whitening, loss of *UCP1* expression	[56]
Correlation with insulin resistance	Positive	Negative	Negative	[54, 56]
Genetic markers	Leptin*,* Adiponectin*,* Resistin*, LPL, G3PDH, ASC1, Tcf21, TLE3, Hoxc8, Hoxc9, Fabp4, PLIN1, Rb1, Rip140, AP2,* Adipsin	*UCP1, Cidea, PPARA, Pgc1a, Prdm16,* citrate synthase, *Eva1, Pdk4, Ebf3, Hspb7, LHX8, Zic1, Epsti1, miR-206, miR-133b, Oplah, Acot2, Fbxo31, Dio2, Cox8b, Ppargc1a, ELOVL3*	*UCP1, Cidea, Pgc1a, PPARA, Epsti1, Tmem26, Cd137, HOX9a, TBX1, CITED1, TNFRSF9, Shox2, Sp100, Ear2, CD40, Dio2, Cox8b, Ppargc2a, Slc27a1*	[33, 37, 38, 40, 50, 56, 59, 60]
Activators	High-fat diet	Cold, exercise, β3-ARs, thyroid hormone, thiazolidinediones, FGF21, BMP7, BMP8B, natriuretic peptides, norepinephrine, meteorin-like, bile acids, adenosine, orexin, irisin	Cold, exercise, thyroid hormone, thiazolidinediones, natriuretic peptides, FGF21, irisin, catecholamines, β3-AR agonists such as CL 316243	[20, 56]
Key transcription factors	Zinc finger protein 423, PPARγ, C/EBPꞵ	C/EBPꞵ, PRDM16, PGC1α, PPARγ, EBF2	C/EBPꞵ, PRDM16, PGC1α, PPARγ, EBF2	[20]

Relative amounts: + (low), ++ (moderate), +++ (high)

Recent studies have shown that beige adipocytes expressing key thermogenic factor UCP1 can develop in WAT in response to certain environmental, genetic, or pharmacological stimuli [40]. These adipocytes were named beige (brite, brown-in-white, brown-like) adipocytes as a group distinct from WAT and BAT [20, 37]. Beige adipocytes arise from sWAT depots in response to β-adrenergic stimulation, diet, or exposure to cold via a reversible process called browning or beiging [20]. However, after the stimulus is withdrawn, beige adipocytes change their expression profile and display white adipocyte features again [41]. Factors such as increasing age, obesity, and metabolic disorders are related to loss of BAT (whitening process) and decreased capacity to induce browning of WAT [20]. Evolutionarily, beige adipocytes are most similar to white adipocytes, as both originate from myogenic transcription factor 5 (Myf5)-negative mesodermal stem cells [37, 42] and functionally, they are most similar to brown adipocytes, as they can convert chemical energy into heat under certain stimuli [41].

Although beige and white adipocytes have the same origin, they appear to have distinct transcriptional profiles and metabolic roles [43]. In mice, beige adipocytes are interspersed within sWAT depots. In adult humans, these adipocytes are located in the cervical and supraclavicular regions [44]. Like brown adipocytes, beige adipocytes are characterized by multilocular /multiple small lipid droplets with variable size, high mitochondrial content, and the expression of thermogenic genes such as *UCP1*, *Pgc1a*, *PPARA*, and *Cidea* [20, 36, 45, 46]. In addition to thermogenic gene expression, beige adipocytes express unique surface markers *PAT2* and *P2RX5* [34, 47] and genetic markers *Epsti1*, *Tmem26* [20, 32, 48], *Cd137* [37], *HOX9a*, *TBX1* [37], *CITED1* [38], *TNFRSF9* [49], and *Shox2* [50]. The principal differences between the three types of AT are summarized in Table 1.

## Origin of white, brown, and beige adipocytes

**Fig. 2:**
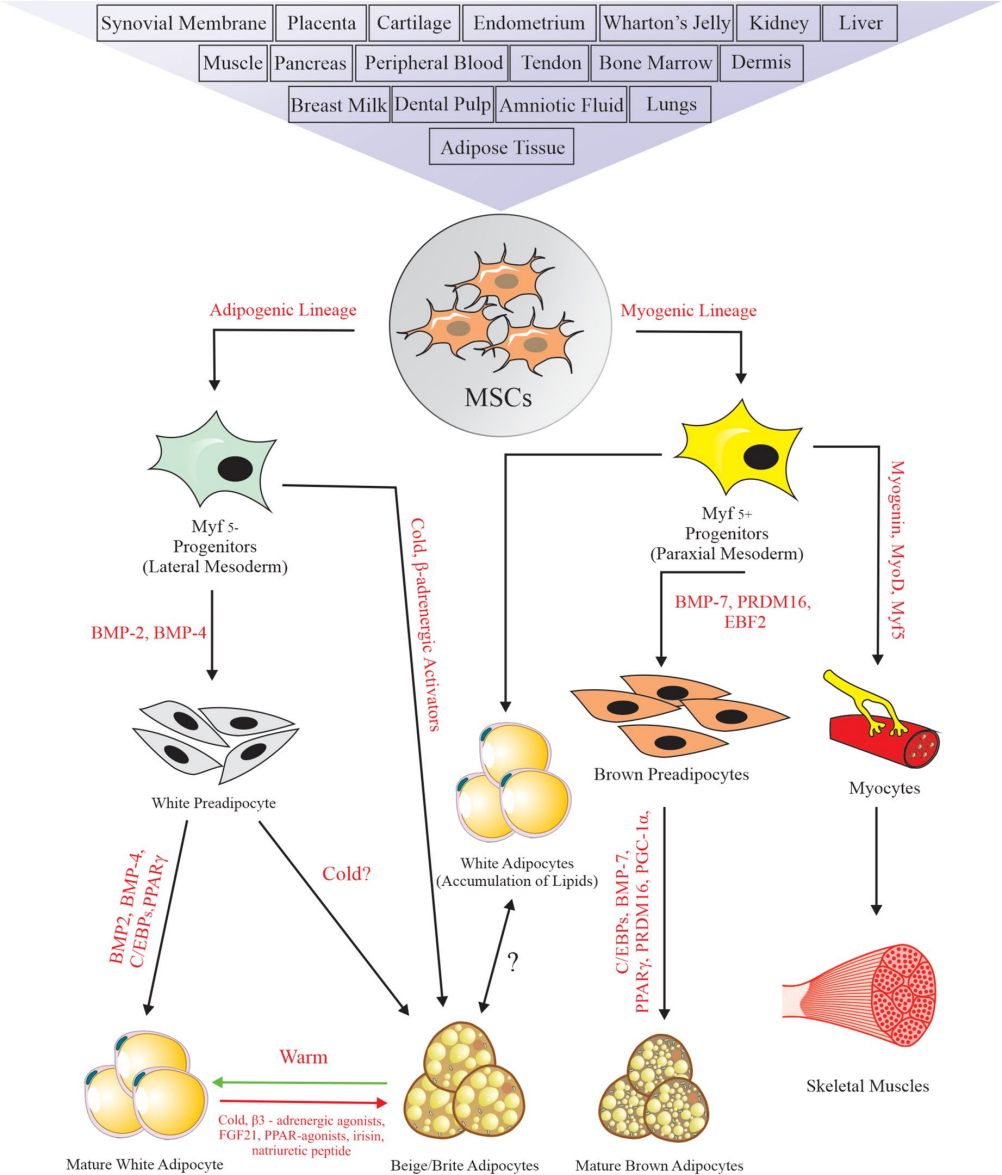
Origin of white, brown, and beige adipocytes. Several sources have been identified for the isolation of mesenchymal stem cells (MSCs), which have a multipotent capacity to differentiate into the mesodermal lineage. The differentiation of MSCs into brown, white, and beige adipocytes is regulated by their essential transcription factors. Brown adipocytes and skeletal muscles are formed from Myf5^+^ progenitor cells. In contrast, white adipocytes are formed from Myf5^+^ and Myf5^−^ progenitor cells. The origin of beige adipocytes is still unknown, but evidence suggests that they are formed from Myf5^−^ progenitor cells or transdifferentiation from mature white adipocytes

The mesenchymal and tissue stem cell committee of the International Society for Cellular Therapy (ISCT) has established standards to describe human MSCs used in research and clinical applications [61]. These standards include (1) the ability of MSCs to adhere to the plastic surface under standard cell culture conditions, (2) multipotency, indicating the capability to differentiate into mature adipocytes, osteoblasts, chondroblasts, and myoblast cells, and expression of cell surface molecular markers such as CD73, CD90 and CD105, while lacking expression of CD11b, CD14, CD19, CD34, CD45, c-kit, CD79a and human leukocyte antigen-DR (HLA-DR). MSCs can be isolated from various tissues, with AT being a particularly abundant and readily available source of mature stem cells that can differentiate along numerous pathways into both adipogenic and myogenic lineages (Fig. 2).

White adipocytes, predominantly with an adipogenic lineage, mainly arise from myogenic transcription factor 5 negative (Myf5^−^) progenitor cells [45]. However, the development of white adipocytes is highly complex, as they can also arise from Myf5^+^ progenitor cells [62]. The adipogenic lineage can be induced by several factors, including bone morphogenetic proteins (BMPs), transforming growth factor-beta (TGF-β), fibroblast growth factor 1 and 2 (FGF1 and 2), insulin-like growth factor 1 (IGF-1), activin, IL-17, and others [62]. Moreover, the expression of transcription factor TCF21, as a unique regulator in preadipocytes, induces differentiation into the white lineage and inhibits differentiation into the myogenic lineage [63, 64]. Brown adipocytes and myocytes, with a myogenic lineage, originate from myogenic transcription factor 5 positive (Myf5^+^) progenitor cells. Myf5^+^ is a main myogenic regulatory factor [45, 65]. The Myf5^+^ progenitor cells turn into brown preadipocytes by BMP7 [66], PR domain containing 16 (PRDM16) [67], and early B-cell transcription factor 2 (EBF2) [68]. Inducing these markers has been shown to result in increased expression of BAT-specific markers, including peroxisome proliferator-activated receptor gamma (*PPARγ*), *PPARγ* coactivator 1 alpha (*PGC1α*), and CCAAT/enhancer-binding proteins (*C/EBPs*) in collaboration with the transcriptional co-regulator PR domain containing 16 (*PRDM16*) [69].

In the ultimate stage of differentiation, preadipocytes are changed into mature adipocytes using specific factors [69, 70]. BMPs are essential for the development of adipocytes. BMP7 induces the expression of the thermogenic protein UCP1, leading to the development of a brown or beige lineage [71, 72]. In addition to the white lineage, BMP4 is also related to the beige lineage, and its excessive expression increases UCP1 levels [73]. β-adrenergic signaling is another essential factor for brown and beige lineage development. PPARγ, a significant regulator of adipogenesis, is involved in the maturation and activity of all three types of adipocytes [64, 74]. It also interferes with β-adrenergic-induced brown adipocyte thermogenesis [75]. PGC1α is a significant regulator of BAT metabolism, which is strongly activated after cold exposure, leading to increased activity of several transcription factors, including PPARs [76]. Additionally, PGC1α enhances fatty acid oxidation, mitochondrial biogenesis, and UCP1 activity in brown adipocytes [76, 77]. The bidirectional cell fate switch between brown adipocytes and skeletal myoblasts is controlled by the transcriptional regulator PRDM16. Key players in the repression or activation of the PRDM16 factor [78] are known to be the transcription factors MyoD and Myf5. The PRDM16-C/EBPβ complex induces *PPARγ* expression in myogenic precursors, activating adipogenic genes in brown adipocytes [79]. Thus, PPARγ, PGC1α, and PRDM16 are three essential factors for achieving the brown adipocyte phenotype [80]. EBF2 controls brown pre-adipose cell identity and is an elective marker of brown and beige adipogenic precursor cells [68, 81].

The source of beige adipocytes is still not clear. Evidence suggests that they may originate from Myf5^−^ progenitor cells or through transdifferentiation from pre-existing mature white adipocytes [82, 83]. White adipocytes can differentiate into beige adipocytes under cold exposure, by β3-adrenergic agonists, PPARγ agonists, FGF21, irisin, and natriuretic peptides [74, 84].

## Secretory factors of brown/beige adipocytes: endocrine, paracrine, and autocrine

**Fig. 3:**
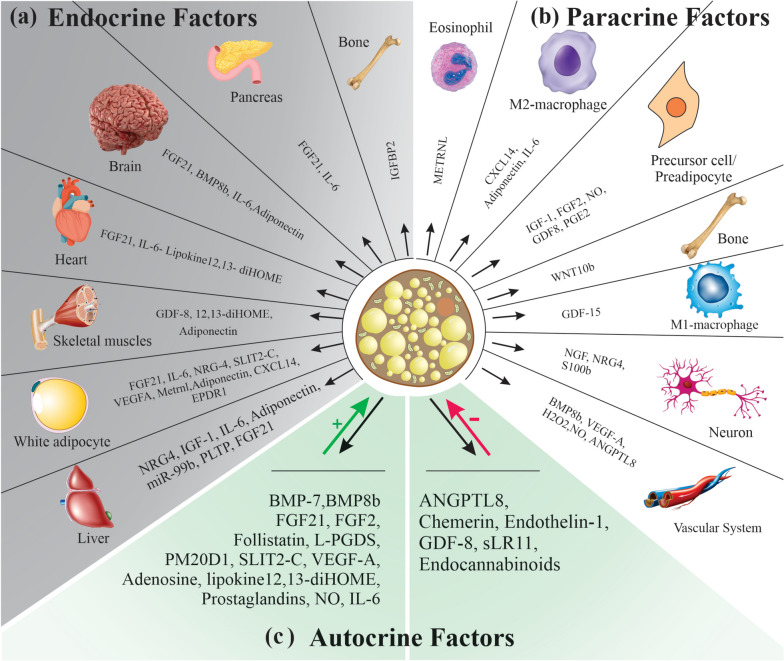
Comprehensive representation of the diverse range of endocrine, paracrine, and autocrine factors secreted by brown/beige adipocytes and their target tissues. **A** Brown/beige adipocytes secrete endocrine factors that may signal distant tissues, including the liver, brain, pancreas, heart, bone, and WAT. **B** Brown/beige adipocytes secrete factors with paracrine roles, which act on the bone, vascular system, nervous, and immune cells contained in BAT and beige ATs. **C** Brown/beige adipocytes secrete peptidic and non-peptidic molecules with autocrine function, leading to a positive (+) or negative (-) effect on BAT thermogenic activity. Factors with a positive function increase mitochondrial activity and the level of UCP1 protein, while factors with a negative function lead to the whitening of BAT, increased lipid droplet size, reduced mitochondrial number, and impaired UCP1 induction

Although research on BAT focuses primarily on its energy expenditure properties as a potential pathway to treat metabolic imbalances, new studies also target the autocrine, paracrine, and endocrine functions of brown/beige adipocytes [85]. Currently, new treatment approaches are focusing on brown/beige adipocytes; identifying the main secretory factors of these cells and characterizing their secretory roles is crucial in discovering novel therapeutic candidates against metabolic disorders such as obesity, type 2 diabetes, and cardiovascular diseases [45, 86]. However, there is still no comprehensive understanding of the brown/beige adipocyte secretomes, and further research in this field is necessary [86]. Regulatory molecules secreted by brown/beige adipocytes, known as brown adipokines or batokines [86], include diverse signaling molecules such as metabolites, lipids, peptides, or microRNAs. These batokines (1) act on the cell that secretes them (autocrine role), which may have positive or negative effects on their thermogenic activity, (2) act on adjacent cells (paracrine role), or (3) are released into the circulation to affect distant cells (endocrine role) [87, 88]. According to several studies from different laboratories, when small amounts of healthy BAT are transplanted into rodent models of obesity and insulin resistance, it improves the recipients' metabolic profiles, weight, and even fecundity [88, 89]. It is likely that factors secreted by BAT may explain their healthy systemic effects. These discoveries highlight the potential of brown adipokines in the treatment of possible metabolic disorders. The following will describe the batokines secreted by brown/beige adipocytes with autocrine, paracrine, and endocrine effects and their tissue targets. The types of these batokines and their tissue targets are shown in Fig. 3.

### Lipokine 12,13-dihydroxy-9Z-octadecenoic acid (12,13-diHOME)

This non-peptidic autocrine factor is secreted by brown adipocytes in response to cold-induced activation of biosynthesis enzymes [90]. 12,13-diHOME operates as a stimulator of BAT activity by increasing the uptake of TGs into brown adipocytes. As a therapeutic agent, this lipokine resolves hypertriglyceridemia in patients with metabolic syndrome and diabetes [90].

### Prostaglandins and prostaglandin-related molecules

Prostaglandins released by brown/beige adipocytes lead to BAT activation and WAT browning. For example, prostaglandin E2, an autocrine factor positively affecting brown/beige adipocyte activity, induces WAT browning in mice and trans-differentiation of human white adipocytes to beige adipocytes [91]. Cyclooxygenase-2 and lipocalin prostaglandin D synthase (L-PGDS) are necessary for prostaglandin synthesis. L-PGDS is produced in BAT and has a positive relationship with BAT activity. The absence of this significant autocrine factor impairs BAT activation and WAT browning [91].

### Adenosine

Adenosine is secreted by activated brown adipocytes via sympathetic responses and, as an autocrine factor, promotes the lipolysis process, which is crucial for thermogenic activation [92]. In addition to its autocrine effects, adenosine causes WAT browning [92].

### Endocannabinoids

Endocannabinoids function as negative autocrine regulators that control the β3-adrenergic pathway-induced BAT thermogenic activation and WAT browning [93, 94].

### Soluble form of the low-density lipoprotein receptor relative LR11 (sLR11)

sLR11, one of the negative regulators of thermogenesis, is derived from brown/beige adipocytes [95]. sLR11 attaches directly to the BMP receptor and thus disturbs its downstream signaling pathway, resulting in a reduced thermogenesis process in a cell-autonomous state [95]. This mechanism has been suggested to minimize extreme energy wastage [95].

### Growth differentiation factor-8 (GDF-8/myostatin)

Following stimulation of starvation-related neural circuits, GDF-8/myostatin is secreted by brown adipocytes [96]. As an endocrine factor, GDF-8 controls the function of skeletal muscles [86, 97]. Moreover, as an essential negative autocrine factor, it regulates WAT browning and BAT thermogenic activity [98, 99]. Myostatin deficiency leads to enhanced differentiation of cells into brown adipocytes and insulin sensitivity [96].

### Follistatin

This soluble glycoprotein batokine is upregulated in brown adipocytes exposed to cold. Unlike GDF-8, follistatin positively affects thermogenic activity [98, 100].

### Bone morphogenetic protein (BMP)

Several BMP members regulate adipocyte differentiation and thermogenesis, influencing white/beige/brown adipogenesis through various mechanisms [43]. BMP7 is mainly generated by stromal vascular cells in AT [66, 101]. Autocrine/paracrine signaling of BMP7 induces the expression of PRDM16 and PGC1α factors in adipose progenitors and leads to the commitment of mesenchymal progenitor cells to brown and beige adipocyte lineages [66, 102]. BMP7 shows significant activity in promoting brown/beige adipocyte differentiation and thermogenesis processes *in vivo* and *in vitro*, and thus, offers a possible new therapeutic approach to treating obesity [66]. BMP4 regulates white and beige adipocyte differentiation [103].

Mature brown adipocytes release the BMP8b batokine in response to noradrenergic-mediated thermogenic stimulus, cold exposure, and nutritional factors such as HFD [104]; it can be used as a novel therapeutic target in obesity and obesity-related disorders. BMP8b promotes BAT responsiveness to β3-adrenergic receptor stimulation and activates p38 MAP kinase signaling through key intracellular effectors such as Smad1, Smad5, and Smad8 [104]. By inhibiting AMP-activated protein kinase (AMPK), BMP8b may have a possible endocrine function in the ventromedial hypothalamus of the brain [104, 105]. BMP8b targets the vascular system through its paracrine action and, with its autocrine action on beige/brown adipocytes, releases neuregulin-4 (NRG4), improving sympathetic innervation [86]. Also, BMP8b is associated with BAT thermogenesis and the regulation of thermogenesis in human newborns [106].

### SLIT2-C

SLIT2-C (a cleaved form of the SLIT2 protein) is an autocrine factor positively affecting BAT activity. SLIT2-C boosts AT thermogenesis by inducing PKA signaling downstream of the β-adrenergic receptor pathway, leading to increased energy expenditure and improved glucose homeostasis [107, 108]. Recently, many studies have shown that circulating SLIT2 levels are negatively correlated with metabolic markers of diabetes mellitus [108]. Besides its autocrine action, SLIT2-C may also have an endocrine effect on the browning/beiging of WAT [107].

### Peptidase M20 domain containing 1 (PM20D1)

PM20D1 is expressed in UCP1^+^ adipocytes and is secreted upon exposure to cold. It catalyzes the biosynthesis of N-fatty acyl amino acids (N-FAAAs) from free long-chain FAs and free amino acids [109]. PM20D1 is involved in energy homeostasis as a UCP1-independent endogenous mitochondrial uncoupler, which increases cellular respiration [109].

### Chemerin

Chemerin is involved in lipid metabolism in an autocrine manner. Its expression in BAT decreases during cold exposure but increases in response to an HFD in mice [110]. Chemokine-like receptor 1 (CMKLR1) and G protein-coupled receptor-1 (GPCR-1), two well-known chemerin receptors, are expressed in brown adipocytes [111].

### Endothelin-1 (ET-1)

ET-1, as an autocrine factor, is released by brown/beige adipocytes. Via induction of the Ednra/Gq signaling pathway, it has a negative effect on thermogenic activity in brown/beige adipocytes [112].

### Vascular endothelial growth factor A (VEGF-A)

VEGF-A is highly expressed in brown adipocytes during cold exposure. VEGF-A promotes macrophage function and induces BAT vascularization by targeting endothelial cells [113]. VEGF-A can also target brown adipocytes as an autocrine factor, essential for BAT activity and thermogenesis [113]. Based on *in vivo* studies performed in obese mice models, a reduced level of VEGF-A leads to the loss of BAT thermogenic potential [114].

### Nitric oxide (NO)

NO generated in brown adipocytes prevents the proliferation and induction of preadipocyte differentiation in primary cultures through NO synthase activities caused by the noradrenergic system [115]. In addition to positive autocrine action, NO plays a paracrine role by targeting vascular cells in BAT [116]. Mineral nitrate also induces NO synthesis, leading to WAT browning [117]. BAT and WAT express metabolically active endothelial (eNOS) and inducible (iNOS) nitric oxide synthases. The deletion of the gene encoding *iNos* improves BAT function [118, 119] and WAT inflammation [120] and metabolism [121] in mice, suggesting a more complex role of nitric oxide in AT metabolism.

### Insulin-like growth factor-1 (IGF-1) and basic fibroblast growth factor-2 (FGF-2)

IGF-1 and FGF-2 secreted by brown adipocytes cause the propagation of preadipocytes in BAT [122]. IGF-1, as a mitogenic peptide, induces the differentiation of brown adipocytes and is mostly secreted by the liver [45]. Thus, IGF-1 acts in an autocrine, paracrine, and endocrine manner [123, 124]. In rodent models of type 1 diabetes, the anti-diabetic activity observed from the transplantation of small amounts of fetal BAT may be due to the secretion of IGF-1 peptide from BAT [124, 125].

### Hydrogen peroxide (H_2_O_2_)

H_2_O_2_ is secreted by brown/beige adipocytes with paracrine effects, acting on vascular cells and suppressing contractility. H_2_O_2_ is produced by the NADPH oxidase-4 enzyme, which is extremely expressed in BAT [126].

### Angiopoietin-like 8 (ANGPTL8)

In response to cold exposure, ANGPTL8, as a secretory regulatory factor, increases in brown adipocytes and suppresses lipoprotein lipase activity bound to the luminal surface of endothelial cells. The function of ANGPTL8 in BAT thermogenic regulation is unknown [127, 128].

### Nerve growth factor (NGF) and S100b

NGF and S100b proteins released by brown adipocytes promote neurite outgrowth upon exposure to cold and enhance sympathetic nerve regeneration in brown and beige ATs [129, 130].

### NRG4

NRG4 is an epidermal growth factor (EGF) family member and is more highly expressed in BAT than WAT. Therefore, it is known as an enriched factor in brown adipocytes [131, 132]. NRG4 is highly induced during brown adipocyte differentiation and enhances in response to β-adrenergic receptor activation in brown adipocytes [133]. NRG4 targets the liver by its endocrine activity, leading to increased oxidation of hepatic fatty acids and suppression of hepatic lipogenesis [131], which has positive effects on insulin sensitivity [45]. Non-alcoholic fatty liver disease (NAFLD) and type 2 diabetes mellitus (T2DM) may result from impaired NRG4 signaling. Therefore, these findings introduce NRG4 as a potential therapeutic target [68, 131]. NRG4 promotes nerve terminal branching through its paracrine action inside BATs [134]. NRG4 has also been suggested to increase the browning/beiging of WAT in response to cold exposure [45].

### Interleukin-6

IL-6 is secreted by BAT in response to noradrenergic stimulation and cold exposure [86]. By paracrine action, IL-6 targets M2-type macrophages and eosinophils; these local immune cells are associated with increased BAT activity and promote WAT browning [87, 135]. Also, IL-6 targets several cell types, including pancreas, brain, liver, and heart cells [136]. IL-6 shows an endocrine action in improving metabolic health and is positively related to obesity, insulin resistance, and type 2 diabetes [137, 138]. IL-6 secretion by beige adipocytes has positive autocrine effects in WAT browning [137].

### Adiponectin

Adiponectin is known as a WAT-derived adipokine. Upon cold exposure, adiponectin is induced in sWAT and promotes sWAT browning via M2-macrophage proliferation. However, it has been reported that adiponectin is also expressed by BAT [45, 139]. In addition to its main function in liver metabolism, adiponectin induces fatty acid oxidation and glucose uptake in skeletal muscles and regulates energy expenditure by affecting the hypothalamus [140].

### Meteorin-like (METRNL)

In response to cold, METRNL is expressed as a circulating hormone by brown/beige adipocytes [141]. This batokine increases the penetration and activation of eosinophils. The production of IL-4 and IL-13 by eosinophils leads to the recruitment of M2-type macrophages [141]. Therefore, by recruiting eosinophils and M2-type macrophages, METRNL also increases BAT activity and WAT browning. It is thus considered as having therapeutic potential in the treatment of inflammatory diseases and metabolic disorders [141].

### Wingless-related MMTV integration site 10b (WNT10b)

WNT10b is secreted by brown/beige adipocytes and has anabolic effects on bone formation [142]. Additionally, WNT10b ameliorates body weight and insulin sensitivity [143].

### Thyroid hormones

Type II thyroxine 5´-deiodinase (DIO2) enzyme changes thyroxine (T4) into an active form of triiodothyronine (T3). DIO2 activity is strongly induced through noradrenergic stimulation. Thus, T3, the first endocrine factor, is crucial for inducing thermogenic activity in brown adipocytes [144].

### Fibroblast growth factor 21 (FGF21)

As a member of the endocrine subfamily of FGFs, the adipo-myokine FGF21 is secreted by BAT and targets several organs and tissues, including the heart, brain, liver, pancreas, and WAT. BAT itself can be targeted through the autocrine actions of FGF21 batokine [86]. The various functions of FGF21 include (1) potent cardioprotective effects, (2) pleiotropic effects on hepatic metabolism and sympathetic outflow, (3) as a potent regulator of metabolic pathways with blood glucose-lowering and insulin-sensitizing effects, (4) control of lipolysis pathway in WAT, and (5) promotion of BAT thermogenesis and WAT browning [45, 145]. The specific tissues affected by FGF21 produced by BAT have not yet been fully characterized.

### Chemokine (C-X-C motif) Ligand 14 (CXCL14)

In response to thermogenic stimuli, the batokine CXCL14 is secreted by brown adipocytes [146]. CXCL14 targets M2 cells or alternatively activated macrophages (AAMs). This function of CXCL14 highlights the ability of batokines to target immune system cells [86]. CXCL14 also boosts WAT browning and BAT activation [146].

### Insulin-like growth factor-binding protein-2 (IGFBP2)

IGFBP2 is known to modulate the functions of IGF-1. IGFBP2 secretion by brown/beige adipocytes may significantly improve metabolic activities and differentiation of preadipocytes [147]. IGFBP2 directly stimulates osteoblast differentiation [148]. This batokine promotes skeletal changes and bone remodeling through its heparin-binding domain. Deletion of the *IGFBP2* gene halts bone turnover, suggesting its potential in addressing skeletal abnormalities. [149].

### Growth and differentiation factor-15 (GDF-15)

Against thermogenic stimuli, GDF-15 is released by brown/beige adipocytes. GDF-15 inhibits the proinflammatory activity of M1-type macrophages through a paracrine role [86, 150]. GDF-15 has been identified as a systemic marker in diseases ranging from cardiovascular diseases to cancers [86]. GDF-15 exerts anorectic effects via its function in the brainstem and controls energy balance [151, 152]. Thus, GDF-15 plays a role in different pathologies (anorexia, obesity, ischemia, and atherosclerosis) by virtue of its varied modes of action. Also, the endocrine role of GDF-15 will be significant in preventing several metabolic disorders [86, 150].

### Ependymin-related protein 1 (EPDR1)

EPDR1 is a newly identified batokine that is released from brown and white adipocytes and is critical for modulating the homeostatic control of energy balance and thermogenic activity of brown adipocytes during the adipogenesis process [153].

### Retinol-binding protein 4 (RBP4)

In mice, RBP4 is expressed in BAT that have been either exposed to cold or treated with PPARγ agonists [154]. Also, *RBP4* gene expression has been shown in brown adipocytes *in vitro* [154]. RBP4 might play a key role in WAT browning [155]. In animal and human studies, RBP4 has been associated with systemic insulin resistance, dyslipidemia, type 2 diabetes, cardiometabolic, and other diseases [156]. However, mechanisms linking RBP4, impaired insulin sensitivity, glucose, and lipid metabolism are still not completely understood [156]. Therefore, further studies are needed to gain insight into the role of RBP4 in the initiation and progression of obesity-related diseases.

### Phospholipid transfer protein (PLTP)

PLTP is a new batokine that targets the liver and increases circulating bile acids, increasing glucose uptake and BAT thermogenesis [157, 158].

### Exosomal microRNAs

BAT activation leads to remarkable shifts in the number of exosomes secreted into circulation and the profile of exosomal miRNAs. Recently, the understanding of BAT has expanded by recognizing the functions of exosomal miRNAs [159, 160]. Specific exosomal miRNAs play a significant role in BAT [161]. For example, miR-92a is an exosomal miRNA that is inversely correlated with the thermogenic activation of BAT and could be applied as a possible serum biomarker to evaluate its functional effect on BAT mass and activity [160]. Additionally, BAT activation leads to the secretion of exosomes containing miR-99b, which exerts hepatic effects by regulating the expression and secretion of FGF21 batokine [159]. Apart from energy expenditure, BAT is believed to modulate body metabolism by regulating other vital organs via exosomal miRNAs. Exosomal miR-132-3p, derived from brown adipocytes, has been shown to downregulate the hepatic expression of lipogenic genes [162]. Hence, the exploration of miRNAs secreted by BAT, WAT, and beige AT, along with a deeper comprehension of their potential functions and specific tissue targets, presents promising avenues for future research. This comprehensive list of miRNAs is further detailed in the following section.

### Other factors

A range of factors are released by brown/beige adipocytes in response to different stimuli, including neuromedin B and nesfatin-1 [163]. Neuromedin B and its receptor are highly expressed in AT. Cikes et al. suggested that neuromedin B does not change body weight and glucose homeostasis on a standard diet nor acts as an insulin‑releasing peptide [164]. Neuromedin B receptor disruption impairs adipogenesis in mice and 3T3-L1 cells. Therefore, neuromedin B receptor antagonism may help limit the increase in adiposity due to pre-adipocyte differentiation [165]. Nesfatin-1, a new depot-specific adipokine, is preferentially expressed in human and murine AT depots. Nesfatin-1 protein expression was markedly increased in high-fat-fed mice and decreased in food deprivation [166]. Also, nesfatin-1 promotes brown adipocyte differentiation by activating the mTOR signaling pathway and may be a promising approach for treating obesity [167]. In BAT, centrally acting nesfatin-1 can induce β3-adrenergic stimulation, an important factor for activating thermogenic genes, releasing heat from interscapular BAT, and ultimately increasing energy expenditure [168].

Brown adipocytes also produce biologically active molecules, such as thermogenic modulators like retinaldehyde and retinoic acid. Upon thermogenic activation, additional secretory factors such as FFAs and lactate are released by these adipocytes [133]. Adipokines secreted by AT participate in the metabolic variations of gestational diabetes mellitus (GDM). GDM is the most common metabolic disorder of pregnancy and has significant consequences for maternal and newborn health. Several studies have shown gene and/or protein expression of adipokines adiponectin, leptin, omentin-1, resistin, IL-1β, IL-6, IL-1RA, IL-10, TNF-α, SOCS3, visfatin, apelin, adrenomedullin, and nesfatin-1) in AT between GDM patients and controls at delivery [169]. Serum visfatin is a metabolic biomarker in obese patients with GDM [170].

Overall, brown and beige adipocytes secrete numerous factors that intricately regulate metabolism. Some stimulate AT activity, enhancing TG uptake and inducing thermogenic activity in brown and beige adipocytes. In contrast, certain factors act as negative regulators, suppressing thermogenesis and inhibiting energy wastage. These factors also interact with immune cells, such as macrophages and eosinophils, influencing BAT activity and promoting browning in WAT. They affect the vascular system, impacting BAT vascularization and influencing the differentiation of mesenchymal progenitor cells into brown and beige adipocytes. Some factors also exert endocrine effects, targeting organs like the liver, pancreas, brain, and heart. They control energy balance, regulate blood glucose, and influence insulin sensitivity. Furthermore, they can recruit immune cells, promote BAT activity, and enhance WAT browning, contributing to metabolic health. In response to various stimuli, such as cold exposure and thermogenic activation, brown adipocytes secrete exosomes containing specific miRNAs. These miRNAs are crucial in regulating BAT mass and activity, affecting energy expenditure and lipid metabolism in different tissues.

## Regulatory microRNAs in adipose tissue

**Fig. 4:**
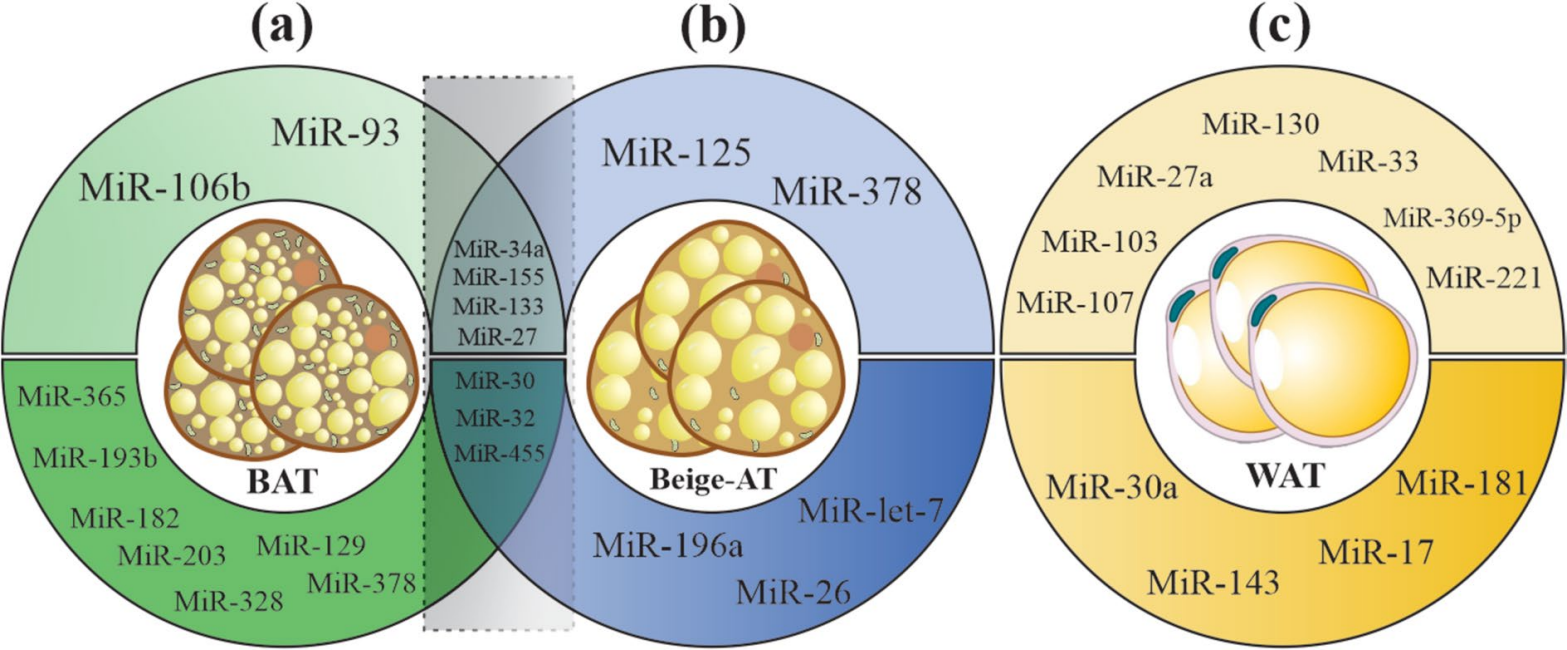
Important regulatory microRNAs in BAT, beige AT, and WAT. **a** Browning inhibiting microRNAs: MiR-106b, MiR-93, MiR-34a, MiR-155, MiR-133, MiR-27. Browning activating microRNAs: MiR-365, MiR-193b, MiR-182, MiR-203, MiR-328, MiR-129, MiR-378, MiR-30, MiR-32, MiR-455. **b** Beiging inhibiting microRNAs: MiR-34a, MiR-155, MiR-133, MiR-27, MiR-125, MiR-378. Beiging promoting microRNAs: MiR-30, MiR-32, MiR-455, MiR-196a, MiR-let-7, MiR-26. Similar MiRs in BAT and beige AT are shown with a light–dark rectangle. **c** Anti-adipogenic microRNAs: MiR-107, MiR-103, MiR-27a, MiR-130, MiR-33, MiR-369-5p, MiR-221. Adipogenic microRNAs: MiR-30a, MiR-143, MiR-17, MiR-181

microRNAs are short, non-coding RNA molecules comprising almost 20–22 nucleotides that take part in RNA silencing and adjust the expression of many genes. miRNAs are necessary for the development, identification, and differentiation of adipocytes. Deleting the *Dicer* enzyme in adipocytes destroys the miRNA processing pathway and ultimately leads to abnormal distribution of adipocytes [159]. microRNAs derived from AT regulate glucose metabolism, thermogenesis, and insulin sensitivity [171, 172]. They have complex relationships with obesity, diabetes, and other obesity-related metabolic diseases such as T2DM and cardiovascular diseases, and they can serve as significant biomarkers for therapeutic purposes. Many microRNAs have been known to regulate important signaling pathways of the adipogenesis process in BAT, beige AT, and WAT by influencing transcription factors that boost or prevent the differentiation of adipocytes [173]. Several microRNAs, such as MiR-193b-365 cluster, MiR-182, MiR-203, MiR-328, MiR-129, MiR-378, MiR-30, MiR-32, and MiR-455, are considered as browning activators, while MiR-106b, MiR-93, MiR-34a, MiR-155, MiR-133, and MiR-27 are known as browning inhibitors (Fig. 4a). Figure 4b shows beiging inhibitor microRNAs (MiR-34a, MiR-155, MiR-133, MiR-27, MiR-125, MiR-378), and beiging promoting microRNAs (MiR-30, MiR-32, MiR-455, MiR-196a, MiR-let-7, MiR-26) in beige AT. An array of microRNAs with adipogenic (MiR-30a, MiR-143, MiR-17, MiR-181) and anti-adipogenic (MiR-107, MiR-103, MiR-27a, MiR-130, MiR-33, MiR-369-5p, MiR-221) properties has been identified in WAT (Fig. 4c) [161, 173–175].

Upon additional energy intake, WAT undergoes dynamic alterations to reflect the equilibrium between energy intake and expenditure. This leads to augmented lipid storage and pre-adipocyte differentiation into mature adipocytes [176]. Pre-adipocyte differentiation is determined by variations in the microRNA expression pattern of WAT, including up-regulated microRNAs (e.g., MiR-103, MiR-107, MiR-143, MiR-26b, MiR-375, MiR-21, MiR-148a, and MiR-30) [173, 177–181] and down-regulated microRNAs (e.g., MiR-221, MiR-155, MiR-210, and MiR-125b) [173, 182, 183]. As the process of adipogenesis in WAT and BAT shows shared pathways, there is an overlap of microRNAs identified in both types of AT but having different effects [184, 185]. EVs, including exosomes and microvesicles, serve as primary carriers transporting microRNAs from producer cells to target cells. Table 2a and b show the functions and targets of each regulatory microRNA in BAT, beige AT, and WAT.

**Table 2:** (a) Key BAT and beige AT derived exosomal microRNAs, their functions, and molecular targets. (b) Key WAT-derived exosomal microRNAs, their functions, and targets

(a)			
**microRNAs**	**Relevant Function (s)**	**Target (s)**	**Reference (s)**
**MiR-193b-365**	Essential regulator for BAT differentiation	*Runx1t1*	[186]
**MiR-182**	Positive regulator of BAT adipogenesis, higher risk of metastasis	*Insig1, Pdgfr2, C/EBPA*	[187, 188]
**MiR-203**	BAT adipogenesis regulation improves insulin sensitivity	*Insig1, Pdgfr2, IFNG*	[189, 190]
**MiR-106b-93**	Negative regulator of BAT differentiation	*Ppara*	[191]
**MiR-328**	Positive regulator of BAT differentiation and adipogenesis regulation	*Bace1*	[192]
**MiR-129**	Positive regulator of BAT function as a potential obesity biomarker	*Igf2, Egr1*	[193]
**MiR-455**	Positive regulator of BAT and beige AT differentiation program, AMPK activation	*UCP1, Tgfbr3,* Necdin*, Runx1t1, Hif1an*	[194–196]
**MiR-30**	As key positive regulators of BAT and beige AT adipogenesis	*Rip140*	[177, 197]
**MiR-34a**	Negative regulators of brown and beige adipogenesis promote obesity-induced adipose inflammation	*Fgfr1, Klf4*	[198, 199]
**MiR-27**	Suppressor of BAT and beige AT adipogenesis, disruptor of brown adipocyte differentiation, and mitochondrial function	*Prdm16, Ppara, Pparg, Creb, Pgc1b,* Prohibitin	[200, 201]
**MiR-378**	Positive regulator of BAT adipogenesis, negative regulator of beige adipogenesis, prevention and treatment of obesity in mice	*Pde1b*	[202]
**miR-133**	Negative regulator of BAT adipogenesis, a suppressor of BAT differentiation	*Prdm16*	[203]
**MiR-155**	An oncogenic miRNA, induced insulin resistance, negative regulator of brown adipogenesis, pro-inflammatory M1-macrophage	*Pparg, C/ebpb, STAT1*	[204–207]
**MiR-32**	Positive regulator of BAT and beige AT adipogenesis	*Tob1*	[208]
**MiR-196a**	Positive regulator of beige AT adipogenesis, induction of WAT browning, regulation of human body fat distribution, a potential therapeutic target to combat obesity and type 2 diabetes	*Hoxc8*	[209, 210]
**MiR-26**	A major regulator of human white and beige adipocyte differentiation, positive regulator of brown adipogenesis	*ADAM17, FBXl19*	[40, 211]
**MiR-125**	Negative regulator of browning (inverse association with UCP1 expression) and formation of functional beige adipocytes	*MMP11*	[212, 213]
**Let-7i-5p**	Inhibitor of BAT thermogenesis, inhibitor of mitochondrial and browning marker genes	Not specified	[214]

(b)			
**MiR-30a**	Stimulator of adipogenesis, improved insulin sensitivity	*STAT1, DLL4*	[215]
**MiR-143**	Regulator of adipogenesis, adipocyte differentiation, and development of obesity-induced insulin resistance	*MAP2K5, ERK5, PPARG, AP2, IGF2R, Orp8*	[216, 217]
**MiR-17**	Improvement of inflammation-induced insulin resistance in macrophages with anti-diabetic activity, a target for T2DM treatment	*ASK1, STAT3*	[218]
**MiR-181**	Promotor of insulin resistance and WAT inflammation, potential novel therapeutic target in the context of obesity	*PTEN, TNFA*	[219, 220]
**MiR-107**	Inhibitor of pre- and mature adipocyte differentiation and lipid accumulation, modulating pre-adipocyte apoptosis, controls insulin sensitivity, potential T2DM biomarker	*CDK6, Wnt3a,* Caveolin-1	[221, 222]
**MiR-103**	Modulator of pre-adipocyte apoptosis, promoter of pre-adipocyte differentiation, a regulator of lipid metabolism and controls insulin sensitivity, potential T2DM biomarker	*Wnt3a, RAI14, MEF2D,* Caveolin-1	[222–224]
**MiR-27a**	Negative regulator of adipocyte differentiation, regulator of hepatic lipid metabolism	*Pparg, FASN*	[225–227]
**MiR-130**	Suppressor of adipogenesis, driving adipocyte differentiation, exhibiting a pro-inflammatory effect	*PPARG,* *Apcdd1*	[228, 229]
**MiR-33**	Negative regulator of pre-adipocyte differentiation, reducer of lipid droplet formation, a regulator of cellular cholesterol efflux and HDL biogenesis	*HMGA2, ABCA1*	[230, 231]
**MiR-369-5p**	Down-regulator of adipogenic differentiation	*FABP4,* Adiponectin	[232]
**MiR-371**	Up-regulator of adipogenic differentiation	*FABP4,* Adiponectin	[232]
**MiR-221**	Negative regulator of adipogenesis, a regulator of inflammation, and insulin sensitivity in WAT	14–3-3γ, *SIRT1, ETS1, AdipoR1*	[233–235]
**MiR-21**	Regulator of adipogenic differentiation	*C/EBPB, TGFBR2*	[236]
**MiR-148a**	Modulator of adipocyte differentiation, a biomarker of obesity	*PTEN, Pparg, C/EBPA, FABP4*	[181, 237]
**MiR-210**	Promoter of mouse diabetic obesity pathogenesis through regulating glucose uptake and the mitochondrial complex IV activity	*Ndufa4*	[238]

## Therapeutic applications

Browning research is a rapidly evolving field with the potential to address a range of metabolic and chronic disorders, such as obesity, diabetes, and cardiovascular disease. Browning agents and nanomedicine-based strategies are two promising therapeutic approaches for browning. Browning agents are compounds that can promote the conversion of WAT into BAT. Nanomedicine-based strategies for browning involve the use of nanomaterials to deliver therapeutic agents to BAT with high precision. This allows for targeted and effective treatment of metabolic disorders. In this section, we will review the latest advances in the development and application of browning agents and nanomedicine-based strategies. We will also discuss the implications of browning for atherosclerosis, cancer, fertility, circadian systems, and disorders.

### Browning agents

Beige adipocytes are a kind of adipocyte found in WAT depots, which can acquire a BAT-like phenotype with an augmented capacity for the thermogenic process, a phenomenon known as WAT browning [239]. The expression of important transcription factors such as *PRDM16*, *PPARA*, and *UCP1* (as a hallmark of thermogenesis) has been observed in WAT [240]. Obesity and diabetes in epidemic proportions are significant worldwide health concerns, and the browning of WAT may hold promise as a target for treating and preventing metabolic disorders, including type 2 diabetes and cardiovascular diseases [241].

A growing number of browning agents [36] have been identified. Table 3 provides a complete list of these agents, extensively evaluated in human and animal models through *in vitro* and *in vivo* experiments. Some of the important browning agents to induce browning in WAT or protect against HFD-induced obesity include cold exposure, β-3 adrenergic receptor agonists (CL 316243, BRL 26830A), short-chain fatty acids (butyrate, propionate, acetate), dietary factors and plant-based compounds (capsaicin, resveratrol, berberine, fish oil, green tea extract, cinnamon, quercetin, curcumin, ginsenoside Rb1), nuclear receptors and ligands (farnesoid X receptor, liver X receptors), microRNAs (microRNA-32, microRNA-455), drug agents (thiazolidinediones, prostaglandin E2, gleevec, β-lapachone, SLIT2-C, artepillin C, adrenomedullin 2), inflammatory factors (IL-6, IL-4, IEX-1), hormonal factors (TH, PTH, PTHrP, GLP-1R, leptin, melatonin, NPs, irisin, FNDC4, maternal secretin), genetic factors (*PTEN, Cox2, Foxc2, folliculin*, *Gq*, and TGF-β/Smad3 pathway), batokines (FGF21, PTGDS, apelin, BMP7, BMP4, NPM3), exercise, PPAR agonists and signaling (rosiglitazone, ABHD6), metabolites (lactate, BHB, BAIBA, retinoic acid), and other factors (gut microbiota). However, the therapeutic use of browning agents [36] is confronted with various obstacles, and strategies to eliminate side effects and/or undesirable metabolic outcomes will be essential.

**Table 3:** WAT browning agents

**Agents**	**Findings obtained from** ***in vitro*** **and** ***in vivo*** **experiments**	**Reference (s)**
**Cold exposure**	Remarkable enhancement in the number of brown adipocytes in the parametrial fat pad of female *BALB/c* mice	[250]
	Increased expression of *Ucp1* in periovarian WAT of rodents in response to + 4 °C	[251]
	Stimulation of insulin signaling pathway in the BAT and muscle of overnight fasted rats	[252]
**β-3 adrenergic receptor agonists:** CL 316243 BRL 26830A	Occurrence of brown adipocytes within traditional WAT depots (mesenteric, inguinal, epididymal, and retroperitoneal) during CL 316243-induced reversal of obesity and diabetes in Zucker *fa/fa* rats	[253]
	Upregulation of *Ucp1* mRNA levels significantly in periovarian WAT depots in rodents	[251]
**Short-chain fatty acids:** Butyrate Propionate Acetate	Butyrate and propionate show protective effects against diet-induced obesity and adjust gut hormones through free fatty acid receptor 3-independent mechanisms in mice models	[254]
	Acetate induces WAT browning and enhancement of hepatic mitochondrial function in *C57BL/6* mice	[255]
**Dietary factors and plant-based compounds:** Capsaicin Resveratrol Berberine Fish oil Green tea extract Cinnamon Quercetin Curcumin Ginsenoside Rb1	Dietary capsaicin stimulates WAT browning to combat obesity in mice	[256]
	Resveratrol increases brown adipocyte development and thermogenic activity in mouse iBAT by promoting the expression of brown adipogenic markers through activation of AMPKα1 in HFD-fed mice. Resveratrol induces brown-like adipocyte phenotype in 3T3-L1 adipocytes by mTOR signaling pathway	[257, 258]
	Berberine significantly induces the formation of brown-like adipocytes in the inguinal WAT depot through AMPK/PGC1α signaling in obese *db/db* mice. Berberine moderates the deacetylation of PPARγ to promote AT remodeling and thermogenesis via the AMPK/SIRT1 pathway and provides a great prospect in obesity treatment	[259, 260]
	Fish oil consumption leads to simultaneous upregulation of *Ucp1* and the β3-AR in inguinal WAT of TRPV1 knockout mice	[261]
	Green tea extract stimulates the expression of browning biomarkers in WAT and restricts weight gain in HED-fed rats	[262, 263]
	Cinnamon significantly increases brown adipocyte markers (*Cidea, Prdm16, Pgc,* and *Cpt1*) expression while reducing white adipocyte markers (*Dpt* and *Igf*) in 3T3-L1 adipocytes and WAT from *db/db* mice	[264]
	Quercetin remodels white adipocytes to brown-like adipocytes in the WAT of mice and 3T3-L1 cells. Also, quercetin reduces plasma TG levels accompanied by WAT browning (increased mRNA expression of *Ucp1* and *Elovl3*) in diet-induced obese *C57Bl/6 J* mice	[265, 266]
	Curcumin causes adipocyte browning and mitochondrial respiratory activity in 3T3-L1 adipocytes and obese rodent model	[267]
	Ginsenoside Rb1 stimulates the browning of 3T3-L1 mature adipocytes through increased mRNA expressions of *Ucp1*, *Pgc1α,* and *Prdm16.* In a dose-dependent manner, ginsenoside Rb1 facilitates the browning of 3T3-L1 adipocytes by repressing the Wnt/β-Catenin signaling pathway	[268, 269]
**Nuclear receptors and ligands:** FXR LXRs	Farnesoid X receptor (FXR) activation with fexaramine antagonist promotes thermogenesis activity and sWAT browning	[270]
	Liver X receptors (LXRs), especially LXRβ, regulate sWAT browning, energy dissipation, and activation of mitochondria	[271]
**MicroRNAs:** MicroRNA-32 MicroRNA-455	microRNA-32 drives BAT thermogenesis and cold-induced sWAT browning in mice	[208]
	microRNA-455 boosts sWAT browning in transgenic mouse models upon cold exposure	[194]
**Drug agents:** Thiazolidinediones Prostaglandin E2 Gleevec β-Lapachone SLIT2-C Artepillin C Adrenomedullin 2	As selective activators of PPARγ, thiazolidinediones such as rosiglitazone induce potent browning of white adipocytes both *in vitro* and *in vivo* via SIRT1, PRDM16, C/EBPα, and PGC1α-mediated mechanisms	[272]
	Prostaglandin E2 acts as a critical regulator of white-to-brown adipogenic differentiation	[273]
	Gleevec, as a PPARγ antagonist, improves insulin sensitivity and induces the browning of WAT and energy expenditure by inhibiting PPARγ phosphorylation	[274]
	β-Lapachone causes WAT browning and increases expression of *Ucp1* through downregulation of miR-382 expression in HFD-fed mice	[275]
	SLIT2-C adjusts beige adipocyte induction through PRDM16 and robust activation of the thermogenic PKA pathway, resulting in increased energy expenditure	[107]
	Artepillin C stimulates brown-like adipocyte formation in murine C3H10T1/2 cells and primary inguinal WAT-derived adipocytes through PPARγ and PRDM16 signaling pathway	[276]
	Adrenomedullin 2 promotes the browning of rat primary adipocytes *in vitro* and decreases obesity and insulin resistance in HFD–fed mice. Adrenomedullin 2, inhibiting the class II MHC in adipocytes, is a suitable candidate for treating early obesity-induced insulin resistance	[277, 278]
**Inflammatory factors:** IL-6 IL-4 IEX-1	In burn injury, an IL-6 signal from bone marrow is essential for WAT browning	[279]
	In response to cold exposure, IL-4 shows a key role in BAT activation in a macrophage-dependent manner	[280]
	Deficiency of immediate early response gene X-1 (IEX-1) induces WAT browning by promoting alternative activation of adipose macrophages and resists diet-induced obesity	[281]
**Hormonal factors:** TH PTH PTHrP GLP-1R Leptin Melatonin Natriuretic peptides Irisin FNDC4 Maternal secretin	Thyroid hormone induces the browning of WAT	[282–284]
	Parathyroid hormone (PTH) and parathyroid hormone-related peptide (PTHrP) cause browning of WAT through activation of the PKA pathway plus energy production via activation of *Ucp1* thermogenic gene	[285]
	Glucagon-like peptide-1 receptor (GLP-1R) agonist (liraglutide) induces BAT thermogenesis and WAT browning through hypothalamic AMPK in mice. Stimulation of brain GLP receptors in the hypothalamus is essential for the activation of BAT thermogenesis and also the browning of white fat	[286, 287]
	Leptin and insulin act synergistically on pro-opiomelanocortin neurons to increase WAT browning	[288]
	Melatonin induces browning of inguinal WAT in Zucker diabetic fatty rats. Melatonin stimulates fat browning by* de novo* differentiation and/or transdifferentiation of white adipocytes	[289, 290]
	Natriuretic peptides promote WAT browning and BAT thermogenic program through several different pathways	[291, 292]
	Irisin is a new adipo-myokine responsible for exercise-induced WAT browning via mitogen-activated protein kinase p38 MAP kinase and ERK MAP kinase signaling. Low-dose irisin induces WAT browning in mice using magnetic resonance imaging	[293, 294]
	Fibronectin type III domain-containing protein (FNDC4), as a new adipokine, decreases lipogenesis and stimulates fat browning in human visceral adipocytes	[295]
	Maternal secretin improves obesity by promoting WAT browning in offspring	[296]
**Genetic factors:** * PTEN* * Cox2* * Foxc2* * Folliculin* * Gq* TGF-β/Smad3	*PTEN*, *Cox2*, *Foxc2*, *folliculin*, and *Gq* genes are associated with WAT browning. *Gq* expression in human WAT is inversely correlated with *UCP1* expression; inhibition of Gq signaling is considered a new therapeutic approach to combat obesity	[112]
	TGF-β/Smad3 signaling pathway activates BAT-like phenotype in rodent WAT	[297]
**Batokines:** FGF21 PTGDS Apelin BMP7 BMP4 NPM3	FGF21 regulates *Pgc1a* expression and WAT browning in adaptive thermogenesis	[298, 299]
	Prostaglandin D synthase (PTGDS) has a positive relationship with BAT activity and WAT browning	[300]
	Apelin, as an adipocyte-derived hormone, increases brown-like characteristics in white adipocytes	[301]
	BMP7 induces the expression of *PRDM16* and *PGC1A* factors in adipose progenitors and leads to the commitment of mesenchymal progenitor cells to brown and beige adipocyte lineages	[66]
	BMP7 and BMP4 induce white-to-brown transition in primary human adipose stem cells from subcutaneous AT	[72]
	Nucleophosmin3 (NPM3), carried by small extracellular vesicles, acts as a batokine that induces WAT browning by regulating the stability of *PRDM16* mRNA	[302]
**Exercise**	Exercise increases *Pgc1a* expression	[77]
	High-intensity exercise is significant for WAT browning and increased FGF21 in skeletal muscle in mice	[303]
**PPAR agonists and signaling:** Rosiglitazone ABHD6	PPARγ agonists induce WAT browning through stabilization of the PRDM16 factor. Rosiglitazone, as a PPARγ agonist, enhances the browning of adipocytes in association with MAPK and PI3-K signaling pathways. Rosiglitazone-loaded nanoparticle increases energy expenditure via WAT browning	[304–306]
	PPARγ signaling includes ABHD6 that negatively adjusts WAT browning	[307]
**Metabolites:** Lactate BHB BAIBA Retinoic acid	Lactate and ketone body beta-hydroxybutyrate (BHB), as strong browning inducers, boost the expression of functional *UCP1* in human and murine WAT through intracellular redox states	[308]
	The adipo-myokine beta-aminoisobutyric acid (BAIBA) enhances the expression of BAT key genes in mouse WAT by PPARα-mediated mechanisms both *in vitro* and *in vivo*. This metabolite also induces BAT-like phenotype in human pluripotent stem cells	[309]
	Retinoic acid stimulates WAT browning by increasing vascular endothelial growth factor signaling and promoting beige adipogenesis of PDGFRα^+^ adipose progenitors	[310]
**Other factors:** Gut microbiota	Gut microbiota modulates both WAT browning and BAT activity. Understanding the relationship between gut microbiota and WAT provides therapeutic strategies against obesity and related metabolic diseases	[311, 312]

### Nanomedicine-based strategies for browning of WAT

**Fig. 5:**
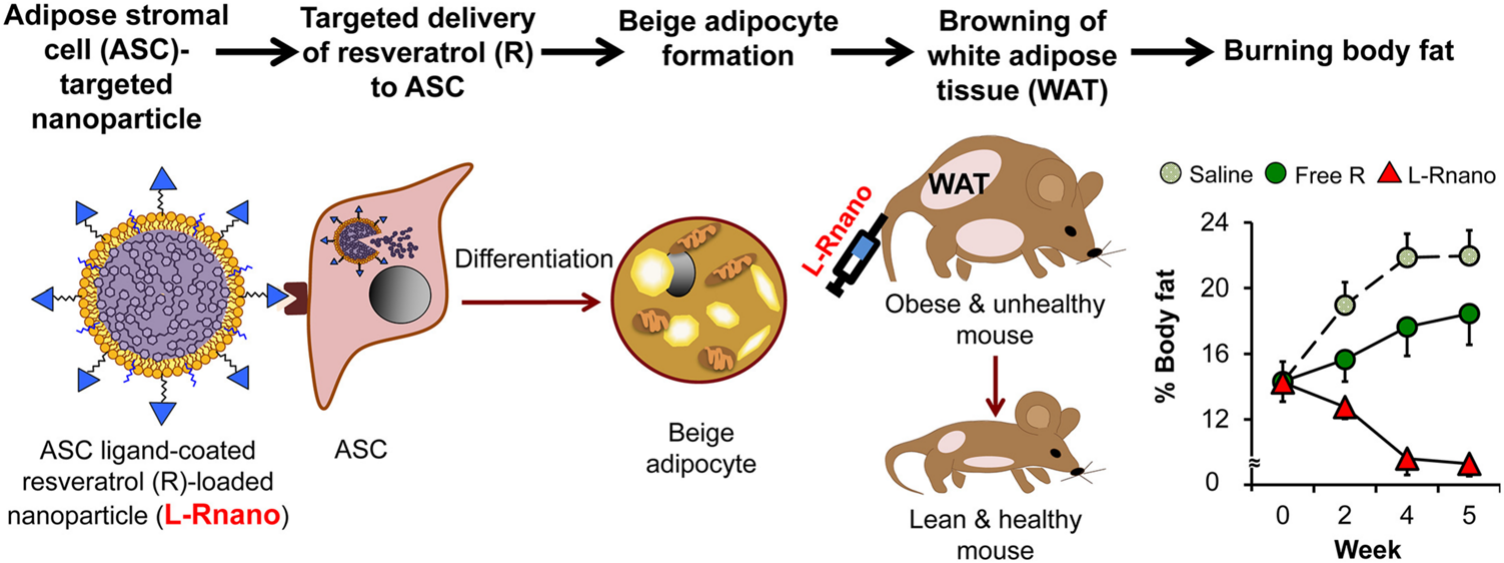
Illustration of L-Rnano designed to deliver R (resveratrol) to ASCs by Zu et al. [243] (with permission). L-Rnano delivers R into the ASCs through delta decorin receptor and stimulates their differentiation into beige adipocytes, subsequently leading to WAT browning, loss of body fat, and ameliorated metabolic health in HFD-induced obese *C57BL/6J* mice

Recent advances in nanomedicine have resulted in the development of various strategies for the targeted delivery of browning agents to WAT. The oral administration or injection of browning agents often leads to undesired side effects, making developing tissue-specific drug delivery systems crucial. Various types of nanoparticles, including poly (lactide-co-glycolide) (PLGA), polyethylene glycol, polyethylenimine, lipid nanoparticles, and hepatitis B core (HBc) protein virus-like particles (VLPs), have been utilized for this purpose [242]. One study using ligand-coated resveratrol-encapsulated nanoparticles (L-Rnano) demonstrated the induction of differentiation of adipose stromal cells (ASCs) into beige adipocytes, subsequently leading to WAT browning, 40% reduction in fat mass, improved glucose and cholesterol homeostasis and decreased inflammation in obese C57BL6/J mice after biweekly intravenous administration for five weeks [243] (Fig. 5). These results indicated that the ASC-targeted nanoparticle delivery system of browning agents could be a promising technology in combating obesity and related metabolic disorders with high efficacy and low toxicity.

Targeted nanoparticles containing rosiglitazone or a prostaglandin E2 analog (16,16-dimethyl PGE2), injected into the vasculature of WAT in mice, were also found to stimulate the browning of WAT and angiogenesis [244]. Transdermal drug delivery systems, such as microneedles and hydrogel patches, have been developed to overcome the challenges of penetrating the skin's intrinsic physiological barrier, resulting in increased delivery efficiency for browning agents [242, 245]. These nanomedicine-based strategies offer great potential for the specific delivery of browning agents to WAT, offering a new method for treating obesity and linked metabolic disorders.

### Browning of WAT as a therapeutic approach to atherosclerosis

Atherosclerosis is a chronic lipid-induced inflammatory disease resulting from atheromatous plaques in medium- and large-sized arteries [246]. Physiological conditions such as cold exposure or β3-adrenergic agonists induce browning of WAT, which leads to an anti-atherogenic profile through the expenditure of FFAs and secretion of adiponectin, FGF21, and apelin [246]. In contrast, in WAT dysfunction in obesity, pro-atherogenic factors, such as FFAs, TNF-α, IL-6, resistin, and leptin, are secreted, which increase the development of atherosclerosis, internal plaque inflammation, and plaque vulnerability [246]. The epicardial AT exhibits a distinct lipid composition and is in close anatomical proximity to the heart tissue [8]. Therefore, WAT browning is considered a potential target in efforts to reduce the expansion of atherosclerotic plaques. Preclinical studies in mice models with preserved hepatic clearance of lipids have shown that browning of WAT by exposure to cold or β3-adrenergic receptor agonists leads to a reduction in the development of atherosclerotic plaques. However, in mice models with impaired hepatic clearance, the development of atherosclerotic lesions and plaque vulnerability is increased [246]. In the mouse model with conserved hepatic lipid clearance, the stimulation of WAT browning promoted by cold exposure, or the use of β3-adrenergic receptor agonists decreases the development of atherosclerotic plaques. In contrast, the growth of atherosclerotic lesions and plaque vulnerability is promoted in the mouse model with impaired hepatic lipid clearance [246]. Clinical studies have shown that people exposed to cold have increased WAT-derived brown-like AT differentiation and have smaller atherosclerotic plaques than people not exposed to cold. However, impaired hepatic clearance of lipids in atherosclerosis has not been investigated in humans [246].

BAT may also exert its effects through indirect mechanisms or by releasing lipid particles that influence systemic metabolism. For instance, in response to cold exposure, murine BAT produces small HDL particles that enhance HDL turnover, potentially affecting cholesterol transport to the liver [247] or modifying HDL's functional composition, which is relevant to atherosclerosis [248]. Recent clinical trials have attempted to detect thinner atherosclerotic lesions in patients with higher BAT activity. Although very few studies have shown a correlation between the induction of WAT browning and atherosclerosis, further research is nonetheless warranted in this exciting field. Strategies to maintain healthy arteries are a serious concern [246]. The study by Shi et al. have revealed that BAT-derived Nrg4 improves atherosclerosis in male mice. Nrg4, as a potential crosstalk factor between BAT and arteries, inhibits endothelial inflammation or adhesion responses, decreases leukocyte homing and macrophage gathering in plaques, improves plaque stability, and thus protects against endothelial injury and atherosclerosis via endothelial ErbB4–Akt–NF-κB signaling pathway. Therefore, Nrg4 may become a novel therapeutic target for atherosclerosis disease [249].

### Brown adipose tissue and cancer

Insulin resistance and chronic inflammation in visceral fat may cause alterations in various molecules, which act on the tumor microenvironment to drive tumor progression [7]. BAT has recently emerged as a potential therapeutic target for cancer. Several studies have proposed that BAT may play a role in cancer-associated cachexia, a condition characterized by muscle wasting and weight loss that is common in cancer patients. However, the complex relationship between BAT and cancer is not yet fully understood. A retrospective study of cancer patients found that BAT was not associated with cachexia or increased mortality [313]. In contrast, another study showed that exosomal miR-155 from gastric cancer cells induced cancer-associated cachexia by suppressing adipogenesis and promoting brown adipose differentiation through C/EPBβ [314]. These findings suggest that the interaction between cancer and BAT may be tumor-specific and context-dependent.

In addition to its potential role in cachexia, BAT has also been implicated in tumor suppression. A recent study showed that cold-induced activation of BAT suppressed tumor growth by inducing a metabolic switch in cancer cells [315]. Another study found that cold-induced brown fat thermogenesis could starve tumor growth [316]. Similarly, a pilot study using FDG PET/CT found a relationship between BAT and breast cancer [317], and a decrease in BAT activity was related to weight gain during chemotherapy in early breast cancer patients [318]. Another study suggested active BAT may protect against cancer cachexia [319].

Overall, these studies indicate that the role of BAT in cancer is complex and may vary depending on the tumor type and stage, as well as other patient-specific factors. More research is needed to fully understand the relationship between BAT and cancer and discover the potential of BAT as a therapeutic goal for cancer.

### Brown adipose tissue and fertility

Numerous studies have identified a connection between disrupted lipid metabolism and fertility issues [320–322]. Pink adipocytes are a female-specific cell type created in the mammary glands during pregnancy, lactation, and post-lactation periods from breast sWAT. Their main function is to secrete milk and store substantial amounts of lipids in the mammary glands during these periods. In the end phase of lactation, pink adipocytes turn into white and brown adipocytes [323]. In addition to its role in metabolism and thermoregulation, BAT has also been implicated in fertility. Several studies have investigated the potential link between BAT, ovarian aging, and male and female fertility. One study suggested that AT dysfunction could contribute to ovarian aging and decreased fertility in women [324]. In particular, the authors proposed that pro-inflammatory cytokines secreted by AT may lead to oxidative stress and inflammation in the ovaries, ultimately impairing ovarian function. However, more research is needed to understand the underlying mechanisms and potential therapeutic strategies for protecting ovarian function.

In male fertility, it has been demonstrated that diet-induced obesity can lead to impaired sperm quality and quantity. However, a mouse model study found that transplantation of BAT from lean mice into diet-induced obese mice improved fertility outcomes [325]. This suggests that BAT may have protective effects on male reproductive function. In a female fertility study using rats for investigating the potential use of rutin, a natural flavonoid, in activating BAT and improving outcomes in polycystic ovary syndrome (PCOS) [326], the authors found that rutin treatment increased BAT activity and improved ovarian function in rats with PCOS. Another rat study demonstrated similar results with cold treatment-induced BAT activation in PCOS [327]. BAT xenotransplantation from rats to mice extended the ovarian lifespan of aging mice, potentially by reducing levels of IL-6 and adiponectin to levels similar to those of young mice [328]. These results suggest that BAT may be a therapeutic target for improving reproductive outcomes in women with PCOS.

Finally, a study investigating the role of BAT in thermoregulation in newborn lambs found that fetal thyroidectomy led to decreased BAT activity and impaired thermoregulation [329]. This highlights the importance of BAT in maintaining proper body temperature and suggests that its dysfunction could negatively affect newborn health. Some of the well-known secretory factors produced by BAT include irisin, FGF21, and adiponectin. These factors have been implicated in regulating insulin sensitivity, glucose and lipid metabolism, and cardiovascular function. Interestingly, recent studies have also proposed that some of these secretory factors may directly or indirectly affect female reproductive function, raising the probability that BAT may be involved in regulating fertility [88, 330]. Recent evidence suggests that the secretome of brown adipocytes, including irisin and leptin, may enhance steroidogenesis in human ovarian granulosa cells, revealing potential mechanisms by which BAT can modulate female fertility [331]. These studies show that BAT may play a significant role in reproductive health and could be targeted for therapeutic applications.

### Crosstalk between adipose tissue, circadian system, and circadian disorders

The circadian timing system governs the 24-h physiological rhythms and adapts the body to environmental changes. Disruptions to this system correlate with diverse health issues, including cancer, sleep disorders, behavioral problems, and metabolic disorders [332–334]. AT plays a central role in the circadian system by influencing human energy regulation. Circadian rhythms regulate adipose processes such as adipogenesis, lipolysis, WAT browning, BAT thermogenesis, and adipokine secretion. Disrupted circadian rhythms due to factors such as aging, shift work, and light exposure at night contribute to cardiovascular diseases and metabolic disorders such as diabetes, and obesity [332–334].

Chronotherapy is a growing field that optimizes treatment by aligning it with human circadian rhythms. It involves timing medications and targeting clock genes using circadian modulators to combat metabolic disorders [333]. Recent research highlights the role of circadian rhythms in human AT dynamics. Studies show that circadian misalignment exacerbates AT reduction in cardiac cachexia, emphasizing the potential benefits of stabilizing circadian rhythms in these cases [335].

BAT influences brain and environmental homeostasis, making it a focus in psychiatry [336]. Light exposure halts BAT thermogenesis, and prolonged daylight correlates with higher human body fat mass [337]. Dysregulated BAT may indirectly contribute to psychiatric disorders and suicide risk [338]. Second-generation antipsychotic drugs, while effective against neuropsychiatric disorders, are linked to metabolic issues, including abnormal weight gain, hyperglycemia, and dyslipidemia, partly due to their effects on BAT and WAT browning [339]. For example, clozapine and lithium lead to inhibition of BAT adipogenesis and weight gain as a side effect [340]. Understanding the intricate interplay between circadian systems, AT, and human metabolic and psychiatric health is crucial and requires further research in this emerging field.

## Conclusion

Although considerable progress has been made in understanding brown/beige adipocyte secretomes, much is still to be learned about their actions and targets. Batokines, regulatory molecules secreted by brown/beige adipocyte secretomes, include a diversity of signaling molecules, such as metabolites, lipids, peptides, or microRNAs. Since microRNAs are thought to play an important regulatory role in critical genes involved in the differentiation and function of WAT, BAT, and beige ATs, their potential to serve as biomarkers in the maintenance of metabolic processes and as targets in the treatment of obesity-related cardio-metabolic diseases cannot be underestimated. Furthermore, the discovery of new browning agents in recent years represents promising approaches to effective therapies for obesity and associated metabolic disorders. Currently available knowledge suggests that further research is warranted on brown/beige adipocyte secretomes and their potential therapeutic applications.

## Data Availability

Data and materials used in this review were obtained from published studies and are available from their respective sources as cited in the reference list.

## Abbreviations

AAMs: Alternatively activated macrophages
ABCA1: Adenosine triphosphate–binding cassette transporter 1
ABHD6: Alpha/beta-hydrolase domain-containing 6
AC: Adenylyl cyclase
ADAM17: ADAM metallopeptidase domain 17
AdipoR1: Adiponectin receptor 1
AMPK: AMP-activated protein kinase
ANGPTL8: Angiopoietin-like 8
aP2: Adipocyte protein 2
APCDD1: Adenomatosis polyposis coli downregulated 1
ARs: Adrenergic receptors
ASCs: Adipose stromal cells
ASK1: Apoptosis signal-regulating kinase 1
AT: Adipose tissue
ATGL: Adipose triglyceride lipase
Bace1: Beta-site amyloid precursor protein-cleaving enzyme 1
BAIBA: Beta-aminoisobutyric acid
BAT: Brown adipose tissue
BHB: Beta-hydroxybutyrate
BMPs: Bone morphogenetic proteins
C/EBPα: CCAAT/enhancer-binding protein alpha
C/EBPβ: CCAAT/enhancer-binding protein beta
C1–4: Complex 1–4
cAMP: Cyclic adenosine 3′,5′-monophosphate
CD36: Cluster of differentiation 36
CDK6: Cell division protein kinase 6
CMKLR1: Chemokine-like receptor 1
CoQ: Co-enzyme Q
Creb: CAMP-response element binding protein
CXCL14: Chemokine (C-X-C motif) ligand 14
Cyto C: Cytochrome C
12,13-diHOME: Lipokine 12,13-dihydroxy-9Z-octadecenoic acid
DIO2: Type II thyroxine 5´-deiodinase
DLL4: Delta-like protein 4
EBF2: Early B-cell transcription factor 2
EGF: Epidermal growth factor
Egr1: Early growth factor response 1
EPCs: Endothelial progenitor cells
EPDR1: Ependymin-related protein 1
ERK5: Extracellular signal-regulated kinase 5
ET-1: Endothelin-1
ETS1: E26 transformation–specific-1
FABP4: Fatty acid-binding protein 4
FAs: Fatty acids
Fasn: Fatty acid synthase
FATP: Fatty acid transport protein
FBXl19: F-box and leucine-rich repeat protein 19
FFAs: Free fatty acids
FGF21: Fibroblast growth factor 21
Fgfr1: Fibroblast growth factor receptor 1
FNDC4: Fibronectin type III domain-containing protein
FXR: Farnesoid X receptor
GDF-15: Growth differentiation factor-15
GDF-8: Growth differentiation factor-8
GDM: Gestational diabetes mellitus
GLP-1R: Glucagon-like peptide-1 receptor
GLUT: Glucose transporter
GPCR-1: G protein-coupled receptor-1
H_2_O_2_: Hydrogen peroxide
HBc: Hepatitis B core
HDL: High-density lipoprotein
HFD: High-fat diet
Hif1an: Hypoxia-inducible factor 1 subunit alpha inhibitor
HLA-DR: Human leukocyte antigen-DR
HMGA2: High-mobility group AT-hook 2
Hoxc8: Homeobox C8
IEX-1: Immediate early response gene X-1
IFNG: Interferon-gamma
IGF-1: Insulin-like growth factor 1
IGF2R: Insulin-like growth factor 2 receptor
IGFBP2: Insulin-like growth factor-binding protein-2
IL: Interleukin
IMM: Inner mitochondrial membrane
Insig1: Insulin-induced gene 1
ISCT: International Society for Cellular Therapy
Klf4: Kruppel-like factor 4
-PGDS: Lipocalin prostaglandin D synthase
L-Rnano: Ligand-coated resveratrol-encapsulated nanoparticles
LXRs: Liver X receptors
MAP2K5: Mitogen-activated protein kinase 5
MCP-1: Monocyte chemotactic protein-1
MEF2D: Myocyte enhancer factor 2D
METRNL: Meteorin-like
MMP11: Matrix metalloproteinase 11
MRI: Magnetic resonance imaging
MSCs: Mesenchymal stem cells
Myf5: Myogenic transcription factor 5
NADR: Noradrenaline
NAFLD: Non-alcoholic fatty liver disease
NDUFA4: NADH dehydrogenase (ubiquinone) 1 alpha subcomplex 4
NGF: Nerve growth factor
NO: Nitric oxide
NPM3: Nucleophosmin3
NRG4: Neuregulin -4
Oplah: 5-Oxoprolinase
Orp8: Oxysterol-binding protein-related protein 8
PCOS: Polycystic ovary syndrome
Pde1b: Phosphodiesterase 1b
Pdgfr2: Platelet-derived growth factor receptor 2
PGC1α: Peroxisome proliferator-activated receptor gamma, coactivator 1 alpha
Pgc1b: Peroxisome proliferator-activated receptor gamma, coactivator 1 beta
PKA: Protein kinase A
PLGA: Poly (lactide-co-glycolide)
PLTP: Phospholipid transfer protein
PM20D1: Peptidase M20 domain containing 1
PPARA: Peroxisome proliferator-activated receptor alpha
PPARγ: Peroxisome proliferator-activated receptor gamma
PRDM16: PR domain-containing 16
PTEN: Phosphatase and tensin homolog deleted on chromosome 10
PTGDS: Prostaglandin D synthase
PTH: Parathyroid hormone
PTHrP: Parathyroid hormone-related protein
RAI14: Retinoic acid-induced protein 14
RBP4: Retinol-binding protein 4
Rip140: Receptor-interacting protein 140
Runx1t1: Runt-related transcription factor 1; translocated to, 1
SIRT1: Sirtuin 1
sLR11: Soluble form of the low-density lipoprotein receptor relative LR11
STAT1: Signal transducer and activator of transcription 1
sWAT: Subcutaneous WAT
T2DM: Type 2 diabetes mellitus
T3: Triiodothyronine
T4: Thyroxine
TG: Triglyceride
TGFBR2: Transforming growth factor beta receptor 2
TGF-β: Transforming growth factor-beta
TNF-α: Tumor necrosis factor-alpha
Tob1: Transducer of ERBB2-1
UCP1: Uncoupling protein 1
VEGF-A: Vascular endothelial growth factor A
VLPs: Virus-like particles
vWAT: Visceral WAT
WAT: White adipose tissue
WNT10b: Wingless-related MMTV integration Site 10b
